# Amyand’s Hernia with Perforated Appendix in a Neonate

**Published:** 2014-09-01

**Authors:** Asif Sandhu, Naeem Liaqat, Sajid Iqbal Nayyar, Rehman Faryal, Shanze Shafique

**Affiliations:** Paediatric Surgery Department, Services Hospital, Lahore

**Keywords:** Amyand's hernia, Appendix, Neonate

## Abstract

When vermiform appendix is found in the inguinal hernial sac, the condition is called Amyand’s hernia (AH). Appendix in hernial sac can be normal, inflamed or perforated. It can present as complicated hernia or acute scrotum. We present a case of Amyand’s hernia in a 25-day-old male who presented with an obstructed hernia having perforated appendix in the hernial sac.

## CASE REPORT

A 25-day-old male patient presented with right inguino-scrotal swelling for 4 days. There was history of bilious vomiting for one day followed by progressive abdominal distension. He was reluctant to feed and had not passed urine for the last 24 hours. Baby was sick and irritable, with high grade fever and signs of dehydration. There was a 2cm x 2cm irreducible tender swelling in right inguino-scrotal region, the upper limit of which was not approachable. The swelling was not trans-illuminant and the testis was not palpable separately. Abdomen was distended and tense with no palpable mass. Bowel sounds were not audible. Radiograph abdomen showed multiple air fluid levels with a cutoff point in right lower abdomen. A provisional diagnosis of obstructed inguinal hernia was made.

Patient was resuscitated and explored. The right inguinal canal was opened through inguinal skin crease incision. On opening of sac, about 3-4 ml of pus was found along with the terminal ileum. On further exploration appendix was found to be perforated at its tip and caecum was hyperemic (Fig. 1). Right testis was viable. Appendectomy was done and contents of the hernial sac were reduced. High ligation herniotomy was done. Postoperative recovery remained uneventful.

**Figure:**
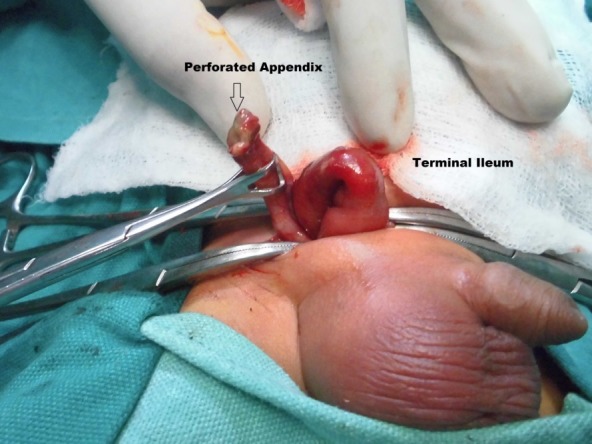
Figure 1:Amyand’s hernia with perforated appendix.

## DISCUSSION

Only few cases of perforated appendix found in inguinal hernial sac are available in literature. AH with perforated appendix can present in any age but discovery in infantile and neonatal age group is extremely rare as in our case. Compression of appendix can hamper its blood supply resulting in perforation, however true pathogenesis is not well established.[1,2] Most cases of AH occur on right side because of the normal anatomical position. Left sided AH are usually associated with congenital anomalies like situs inversus and malrotation. Preoperative diagnosis of hernial appendicitis/ perforated appendicitis is very difficult.[3] In case of non-inflamed appendix only herniotomy is recommended while with the evidence of acute inflammation, trans- hernial appendectomy should be done. A high mortality rate ranging up-to 30% is reported especially in the cases of perforated appendicitis with or without peritonitis.[4] AH with appendicitis is very rare, especially in neonates. A high index of suspicion is required to keep this rare condition in the list of differential diagnoses of children with irreducible inguino-scrotal swelling.

## Footnotes

**Source of Support:** Nil

**Conflict of Interest:** None declared

